# Hypertension in Syrian refugees: prevalence, awareness, and access to care in Denmark and Lebanon

**DOI:** 10.3389/fpubh.2025.1486806

**Published:** 2025-06-19

**Authors:** Tala Al-Rousan, Wadih Naja, Christian Morberg Wejse, Dahlia Kaki, Michaelangelo Aoun, Saskia Lange, Laith Alshawabkeh, Reema Alhamad, Nadine Kadri, Andreas Halgreen Eiset

**Affiliations:** 1 Herbert Wertheim School of Public Health, University of California, San Diego, San Diego, CA, United States; 2 Department of Psychiatry and Clinical Psychology, Faculty of Medicine, University of Balamand, Beirut, Lebanon; 3 Department of Psychiatry and Clinical Psychology, Mont Lebanon Hospital - University Medical Center, Hazmieh, Lebanon; 4 Department of Psychiatry, Lebanese University, Beirut, Lebanon; 5 GloHAU, Center for Global Health, Department of Public Health, Aarhus University, Aarhus, Denmark; 6 University of California, San Francisco, San Francisco, CA, United States; 7 Department of Biomedicine, Aarhus University, Aarhus, Denmark; 8 Charité - University Medicine Berlin, Berlin, Germany; 9 Division of Cardiology, Department of Medicine, University of California, San Diego, San Diego, CA, United States; 10 University of Waterloo, Waterloo, ON, Canada; 11 Western University of Health Sciences, Pomona, CA, United States; 12 Department of Clinical Pharmacology, Aarhus University Hospital, Aarhus, Denmark; 13 Department of Anesthesiology, Regional Hospital Horsens, Horsens, Denmark

**Keywords:** hypertension, refugees, healthcare access, prevalence, global health

## Abstract

**Background:**

Hypertension significantly contributes to global morbidity and mortality rates. Refugees are often exposed to significant risk factors that increase their chances of developing hypertension; however, the prevalence of hypertension among resettled refugees remains understudied. This study estimates and compares the prevalence of hypertension, levels of awareness, and access to care between two groups of Syrian refugees residing in humanitarian settings in Lebanon and Denmark.

**Methods:**

We analyzed cross-sectional data collected from refugee camps in Lebanon and asylum centers in Denmark. Cluster-randomized sampling was conducted between January 2016 and December 2019. We present the estimates of hypertension prevalence, psychosocial stress, and lifestyle factors by strata, along with prevalence differences adjusted for confounding using propensity score weighting. Multiple imputation was employed to address missing data, and 95% bootstrap confidence intervals were reported.

**Results:**

Of the 712 participants, 113 were from Denmark and 599 from Lebanon. Among them, 68% were women, and the median (IQR) age was 34 years (19). The self-reported prevalence of hypertension was 20% in Lebanon and 11% in Denmark. The prevalence of measured stage 2 hypertension was 23% in Lebanon and 24% in Denmark. The confounder-adjusted and multiple-imputed prevalence of hypertension was 5.0 (95% CI: −4.7; 16.1) percentage points higher in Denmark than in Lebanon. Among those with a known diagnosis of hypertension, only 1.6% in Lebanon and none in Denmark were currently taking medications for hypertension. The median (IQR) number of days since last seen by a medical professional was 8 days (48) in Lebanon and 8 days (21) in Denmark. The mean body mass index (BMI) was 27.0 (SD = 6.15) in Lebanon and 26.2 in Denmark (SD = 6.53). In Denmark, 50.5% reported smoking, and 23.6% reported drinking alcohol, while in Lebanon, the figures were 27.4 and 0.5%, respectively.

**Conclusion:**

Hypertension prevalence was found to be high among refugees, even in high-income countries (HICs) such as Denmark. Lack of access to medications in relation to self-reported prevalence was observed in both Lebanon and Denmark. Further investigation into psychosocial stress in humanitarian settings as a contributing factor to hypertension disparities in this population is crucial.

## Introduction

Hypertension is a leading modifiable cause of premature death (1). In the past decade, prolonged armed conflict has resulted in unprecedented numbers of forcibly displaced populations, with Syrians comprising the largest refugee population globally (2, 3). High-stress situations, such as war and migration, are potential risk factors for hypertension. One large population-based study reported that people deployed to war zones were 1.3 times more likely to report hypertension (4). Currently, the number of displaced people, including refugees and asylum seekers, is at an all time high. The United Nations High Commissioner for Refugees reported that 1.5 million Syrian refugees live in Lebanon and Denmark, which are top asylum-seeking destinations in general and home to 35 thousand Syrian refugees and asylum seekers.

Refugees have a higher risk of cardiovascular disease (CVD) compared to host populations and other migrant groups, but there is limited literature on CVD risk factors, such as hypertension, among refugees (5, 6). In fact, many studies have called for more research on hypertension prevalence among refugees (7). One scoping review, for example, highlighted the variation in hypertension prevalence rates by host country and country of origin, suggesting differences in population characteristics, host country refugee health systems, and study methodologies, and emphasized the need for further research (8). Numerous factors contribute to the prevalence of non-communicable diseases (NCDs) among refugees, including specific risk factors encountered during their migration. Many refugees walk on foot for days to reach a safe place in neighboring low- and middle-income countries (LMICs), such as Lebanon, or embark on dangerous journeys to reach high-income countries (HICs), such as Denmark. Resettlement of refugees often involves humanitarian organizations scrambling to provide basic needs. During this time, host country protocols often focus on episodic care to prevent disease outbreaks and reduce mortality. As a result, NCD screening is not routinely conducted during health assessments (9). The health screenings offered to refugees after resettlement varies by location, resulting in vast differences in which health conditions are screened and who can access these services (10). In addition, refugees face multiple barriers to healthcare. Many are unaware of their legal rights to healthcare or are afraid to disclose conditions that would jeopardize their immigration process (11). Language, cultural, and other logistic barriers also affect healthcare-seeking behaviors (12).

Consistently, hypertension has been the most prevalent NCD among Syrian refugees resettled in LMICs, but research on this issue remains limited in HICs (13, 14). Proper management of hypertension requires identifying factors related to forced displacement that influence risk, awareness, and access to care along the care continuum throughout the migratory route. Given the current difficulty of estimating hypertension prevalence in Syria, this study focuses on understanding the epidemiology of hypertension among Syrian refugees in two distinct and major migration stations: Lebanon and Denmark. As part of the Asylum seekers’ and Refugees’ Changing Health (ARCH) study—designed to assess the health impact of the migration process, living conditions, access to healthcare, and health transitions—we conducted interviews and clinical assessments of Syrian refugees who have recently resettled in Lebanon and Denmark (15). This study aimed to quantify the prevalence of hypertension, determine the proportion of known and unknown diagnoses of hypertension, and evaluate access to care for hypertension in the refugee camps.

## Methods

### Population

In Lebanon, Syrians are referred to as “refugees” because Lebanon’s stressed immigration systems stopped processing asylum applications in 2015 and migrants could not be returned to Syria. In Denmark, Syrians are referred to as “asylum seekers” and face the risk of deportation until their asylum case is reviewed with a positive outcome. Lebanon ranks third globally in the number of refugees per capita and hosts more than one million Syrian refugees. Refugees in Lebanon live in accommodations ranging from regular (though often dilapidated) buildings to improvised tents clustered in informal gatherings and formal refugee camps scattered across the country. In 2017, approximately half of these accomodations did not meet minimum humanitarian standards (16). Additionally, Lebanon has faced inflation and multiple internal crises in recent years (17).

### Data collection and participant recruitment

We used a cluster sampling design to recruit participants between January 2016 and December 2019. Supplementary Table 1 provides the estimated source population and the target sample size of 1,100 in Lebanon. To implement stratified cluster sampling with proportional allocation in Lebanon, local non-governmental organization partners assisted in stratifying the country into five regions—Beirut, North, South, Bekaa, and Mount Lebanon—based on geography, infrastructure, and local concerns. All gatherings, rural communities, and settlements including formal and informal refugee camps of Syrian refugees were identified, and the included sites were selected randomly within each geographic cluster. All eligible individuals within these sites were invited to participate. In Denmark, all five asylum center operators were invited to participate in the study; three of the five operators accepted to participate, representing 11 accommodation centers. After consultation with local and international NGOs, six centers were randomly selected for participation. All eligible asylum seekers at these centers were invited to participate, with the goal of including 220 individuals and covering the majority of locations proposed by the consulted NGOs. Justification for the target sample size is provided in a previous study (15). The implemented inclusion criteria were as follows: (a) adult (≥ 18 years old), (b) born in Syria, (c) left Syria after the onset of the war (February 2011), (d) arrived in the host country less than 12 months before inclusion, and (e) residing at the inclusion site at the time of the study. The exclusion criteria included any physical or mental illness that prevented participation or impaired the capacity to provide informed consent.

### Measures

We investigated the association between the host country and hypertension prevalence rates. Hypertension was diagnosed based on the patient’s medical history, inferred from their medication list (taking or being prescribed antihypertensive medication), or determined by measuring BP with a systolic reading of ≥140 mmHg or a diastolic reading of ≥90 mmHg. Blood pressure (BP) was measured once using a recently calibrated, hospital-grade automatic sphygmomanometer, after at least 5 min of rest in a seated position, with the cuff on the right arm at chest height. This was performed as part of a clinical examination, which also included a thorough medical history. Relevant variables to address bias were identified *a priori* after discussions among the authors, guided by a graphical representation of our assumptions (Supplementary Figure 1). These variables included age, sex, socioeconomic status, medication, and substance use and were collected using a questionnaire that was developed, translated, and back-translated between Danish and Arabic by independent bicultural translators. The final questionnaire was approved after minor adjustments by authors MPA and WJN and piloted on 10 participants recruited from the Danish asylum centers. This also led to minor adjustments, including clarifications in the explanatory texts supplementing some questions and changes to the order of questions. In addition, waist circumference, weight, and height were measured as part of the clinical examination, and body mass index (BMI) was calculated as weight in kilograms divided by squared height in meters. Obesity was defined as a BMI of ≥ 30. Finally, psychiatric comorbidity was assessed using the World Health Organization (WHO)-5 Well-Being Index, with a score of < 13 indicating poor mental health and suggesting the need for psychiatric evaluation (18, 19). All other comorbidities were identified through the medical history or inferred from the medication list (e.g., antidiabetic medicine, cholesterol-lowering medicine, etc.). Variables collected but not utilized in the present study are listed in the study protocol (15).

### Randomization and inclusion

In this study, data collectors were either healthcare professionals or students. They obtained informed oral and written consent from potential participants, assisted those who could not read or write by addressing any clarifying questions, gathered their medical history, and conducted examinations with the assistance of a certified interpreter. The questionnaire was completed independently. Healthcare referrals were secured for those who needed immediate somatic or psychiatric evaluation. Individuals who declined to participate were asked to provide basic demographics and reasons for refusal. The sampling frame, sample size estimation for each region, and training of the data collection team are detailed in the study protocol (15).

### Statistical analysis

Our applied statistical method and methodological considerations and implications are described in previous publications (20, 21). Estimates of hypertension prevalence were calculated to assess the known disease burden, using the number of individuals with hypertension as the numerator and the total size of the population as the denominator (22, 23). The propensity score-weighted prevalence difference of hypertension between Lebanon and Denmark was estimated using “standardized mortality ratio” weights to address confounding effects. The propensity score model for the analysis was selected from three proposed exposure models of increasing complexity (Supplementary Figure 2; Supplementary Table 2). Each model was examined using three levels of extreme weight truncation: none, at the 1st and 99th percentiles and 5th and 95th percentiles. It was decided *a priori* to use the simplest model with the least truncation, provided that all covariates were balanced between exposure groups (i.e., absolute standardized mean differences were ≤10%). Multiple imputation was used to handle missing data. For each partly observed variable, where the missingness mechanism was considered “everywhere missing at random,” missing values were imputed using the substantive model and additional auxiliary variables that were known or empirically shown to be predictors of the variable in question, resulting in a response-and-predictor matrix. Due to modeling in both the propensity scores and multiple imputation, a 95th percentile bootstrap confidence interval (95% CI) was produced. Sensitivity analyses were performed to assess the model choice (using a more complex propensity score model that also achieved acceptable balance), to evaluate the chosen threshold for hypertension (by repeating the analysis with increased thresholds: systolic BP ≥ 150 mmHg and diastolic BP ≥ 100 mmHg), and to examine the impact of multiple imputation (three complete case analyses were carried out: with no adjustment, adjusting only for age and sex, and using the same propensity score model as in the main analysis) (see Supplementary Table 3). All data management, analysis, and plotting were performed using R software (v4.2.2), with particular use of the “Tidyverse” packages for data management and plots (24), “smcfcs” for multiple imputation (25), “WeightIt” for obtaining propensity score weights (26), “boot” for parallelized bootstrapping (27), and “furrr” for further parallelizing (28).

## Results

**Figure 1:**
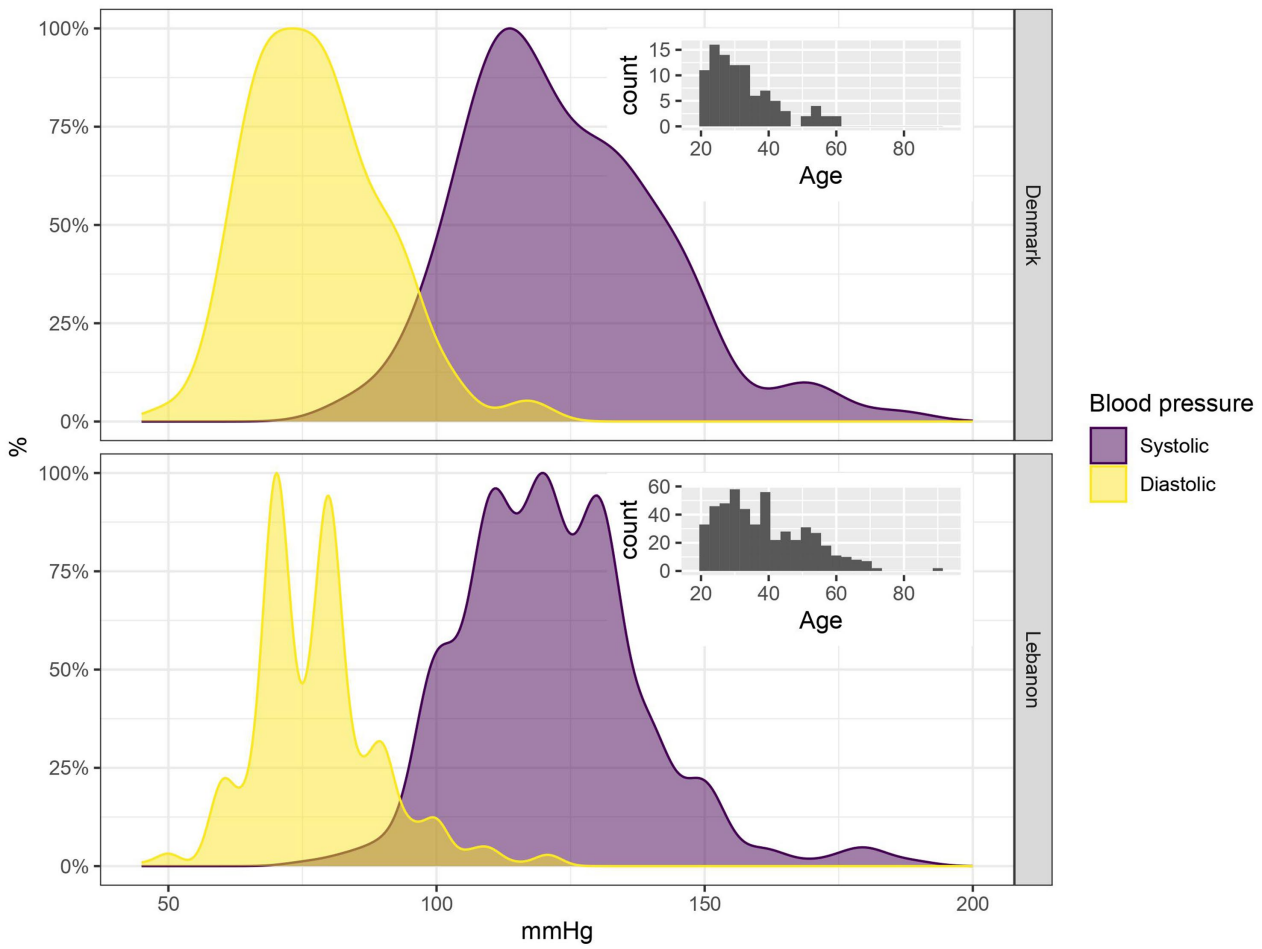
Blood pressure distribution by host country. Distribution of blood pressure and age in Denmark and Lebanon. On average, the study population in Lebanon had higher blood pressure and was also slightly older than the population in Denmark. The bi- and triphasic appearance of the distribution in Lebanon may be ascribed to “the rounding effect”.

Table 1 presents the characteristics of the entire sample from both Lebanon and Denmark. On average, the study population in Lebanon was older than those in Denmark, but BP remained comparable between both groups (Figure 1; Tables 1, 2). Of the 712 participants, 23.6% (*n* = 155) presented with elevated BP levels (23% in Lebanon, 24% in Denmark) (Table 2).

**Table 1:** Characteristics of the study sample.

Characteristic	All *N* (%)	Refugees in Lebanon (*N*%)	Refugees in Denmark (*N*%)
Invited	751	630	121
Refused to participate	39 (5.2%)	31 (4.9%)	8 (6.6%)
Included	*N* = 712 (100%)	*N* = 599 (100%)	*N* = 113 (100%)
Age group			
18–29	225 (34.9%)	175 (32.3%)	50 (48.5%)
30–39	186 (28.9%)	153 (28.3%)	33 (32.0%)
40–59	193 (30.0%)	175 (32.3%)	18(17.5%)
≥60	40 (6.2%)	38 (7.0%)	<5 (<5%)
Missing	68 (9.6%)	58 (9.7%)	10 (8.8%)
Female	461 (68.4%)	408(72.7%)	53(46.9%)
Self-rated SES before migration			
Below average	155(25.4%)	137(25.5%)	18 (24.7%)
On average	378(61.9%)	341(63.4%)	37 (50.7%)
Above average	26(4.3%)	24(4.5%)	<5 (<5%)
Do not know	52(8.5%)	36(6.7%)	16 (21.9%)
Missing	101 (14.2%)	61 (10.2%)	40 (35.4%)
Marital status			
Single	92(13.6%)	59 (10.4%)	33 (30.3%)
Married/Living with a partner	534(78.8%)	465 (81.7%)	69 (63.3%)
Widowed	41(6.0%)	35 (6.2%)	6 (5.5%)
Other	11(1.6%)	10 (1.8%)	<5 (<5%)
Missing	34 (4.8%)	30 (5%)	<5 (<5%)

**Table 2:** Hypertension prevalence and cardiovascular disease factors among the Syrian refugees in the humanitarian settings in Lebanon and Denmark.

CVD factor	All *N* = 712 *N* (%)	Refugees in Lebanon *N* = 599 *N* (%)	Refugees in Denmark *N* = 113 *N* (%)
BP systolic (mm Hg), mean (SD)	122 (17.1)	122 (16.8)	124 (18.6)
BP diastolic (mm Hg), mean (SD)	77.7(11.7)	78 (11.6)	78 (12.5)
Hypertension, measured (systolic BP ≥ 140 or diastolic BP ≥ 90 mm Hg)	155 (23.7%)	128 (23.6%)	27 (24.5%)
Among those with measured hypertension: reporting having hypertension	34 (21.9%)	32 (25.0%)	≤5 (≤18.5%)
Among those reporting hypertension: taking antihypertensive medications	9 (1.4%)	9 (1.6%)	0 (0.0%)
Days since last seen by a medical professional, median (IQR)	8 (37)	8 (48)	8 (21)
BMI (kg/m2), mean (SD)			
All	26.9 (6.2)	27.0 (6.2)	26.2 (6.5)
Female	27.5 (6.6)	27.5 (6.3)	27.9 (8.1)
Male	25.4 (5.0)	25.7 (5.3)	24.6 (4.2)
BMI class			
Underweight	20(3.1%)	17 (3.2%)	<5 (<5%)
Normal	262 (40.9%)	213 (39.8%)	49 (47.0%)
Overweight	202 (31.6%)	166 (31.0%)	36 (34.3%)
Obese	156 (24.4%)	139 (25.9%)	17 (16.2%)
Diabetes Mellitus			
Self-reported diagnosis	61 (8.6%)	57 (9.5%)	<5 (<5%)
Taking anti-DM medications, among those reporting having DM	8 (1.1%)	6 (1.0%)	<5 (<5%)
Current smokers	210 (31.1%)	155 (27.4%)	55(50.5%)
Alcohol consumption	28 (4.2%)	<5 (<1%)	25 (23.6%)
Waist circumference >102 cm			
All	170 (26.4%)	149(27.5%)	21(20.5%)
Female	121 (28.6%)	106 (28.5%)	15 (29.4%)
Male	36 (19.3%)	30 (22.1%)	6 (11.8%)
History of CVD (self-reported or previous MI)	53 (7.4%)	47 (7.8%)	6 (5.3%)
WHO-5 score, median (IQR)	24 (40)	24 (32)	24 (48)
WHO-5 score < 50^a^	515 (77.9%)	437 (79.2%)	78 (71.6%)
WHO-5 score < 13^b^	200 (30.3%)	166 (30.1%)	34 (31.2%)

DM, diabetes mellitus; CVD, cardiovascular disease; MI, myocardial infarction; IQR, interquartile range; WHO, World Health Organization.

^a^Indicates risk of clinically relevant depression or other psychiatric condition.

^b^Indicates poor mental well-being. Further psychiatric evaluation is recommended.

**Figure 2:**
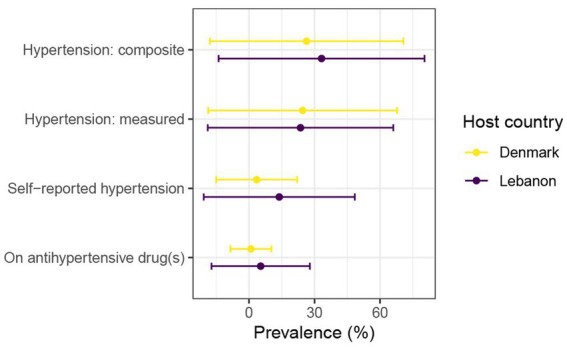
Prevalence with standard deviation by host country. The composite measure categorized an individual as having hypertension if one or more of the following criteria were met: self-reported diagnosis of hypertension, use of antihypertensive drug(s), or measured blood pressure ≥140/90 mmHg.

Among those with elevated BP levels, 21.3% (*n* = 33) reported a known diagnosis of hypertension (25% in Lebanon and < 19% in Denmark). Only nine participants (all from Lebanon) who had hypertension also reported taking antihypertensive medications. The prevalence of hypertension according to the different indicators is shown in Figure 2.

The median number of days since last seen by a medical professional was 8 (IQR = 48) in Lebanon and 8 (IQR = 21) in Denmark. The distributions of both BMI and waist circumference were comparable between Lebanon (27 kg/m2 and 93 cm) and Denmark (26 kg/m2 and 94 cm). The proportion of obese participants was higher in Lebanon (25.9%) compared to Denmark (16.2%). Smoking and alcohol consumption were more widespread in Denmark (50 and 24%, respectively) compared to Lebanon (27 and <1%, respectively). The WHO-5 results showed low levels of mental well-being among all participants, with 77.9% (*n* = 515) scoring below 50 points. The proportion of participants with a low score was slightly higher in Lebanon (79.1%) compared to Denmark (71.5%). Approximately one-third of the participants in both Lebanon (*n* = 166) and Denmark (*n* = 34) scored below 13 points.

After multiple imputation and adjustments for confounding, the prevalence of hypertension was found to be 5.0 (95% CI: −4.7; 16.1) percentage points higher in Denmark than in Lebanon. In the sensitivity analysis, the fully adjusted model produced a slightly lower estimate of the prevalence difference (4.2 [95% CI: −4.4; 13.5] percentage points) (Supplementary Table 3). This finding was robust to the threshold for measured hypertension: when the threshold BP was set to ≥150/100, the estimated prevalence difference decreased marginally to 4.0 (−4.7; 13.8) percentage points. The missingness mechanisms were considered “everywhere missing at random” for all partly observed variables. The multiple imputation produced datasets that resembled the original data for all key variables (Supplementary Table 4). The complete case analyses returned lower estimates and, in two cases, effects in the opposite direction.

## Discussion

This cross-sectional, multi-site study documents the prevalence of hypertension and disease awareness among recently displaced Syrian refugees and asylum seekers in Lebanon and Denmark. Although comparative data from Syria are non-existent, the hypertension prevalence rates in Lebanon and Denmark were comparable and lower than those reported in other LMICs such as Jordan and Turkey (7, 29). The prevalence rates in this sample were also lower than those in local populations in Lebanon and comparable to the Danish population in Denmark (30, 31). A smaller proportion of individuals with hypertension in Denmark were aware of their diagnosis compared to their counterparts in Lebanon, suggesting poorer health status awareness in Denmark. Together with the evidence of a growing burden of hypertensive health disparities among refugees who resettled years ago, these findings offer a comprehensive picture highlighting a missed opportunity for addressing the global hypertension disease burden in the growing refugee population (1, 29, 32, 33).

The prevalence of hypertension among the Syrian refugees was 5.0 percentage points higher in Denmark compared to Lebanon. It is well-documented that mental distress is disproportionately prevalent in asylum shelters (34). The high prevalence of smoking and alcohol consumption among asylum seekers in Denmark indicate that refugees in Denmark may experience more psychosocial stress levels and poorer cardiovascular outcomes, if left unmanaged. Given that the initial stages of resettlement are particularly critical and that asylum seekers in Denmark are not allowed to work, offering psychosocial support can alleviate such stress in asylum seekers—particularly benefiting young adults, who are at increased risk of cardiovascular disease and all-cause mortality if they develop hypertension early (35, 36).

These findings call for further research to specifically examine the effects of living conditions and psychosocial stress in settings such as refugee camps, migrant detention centers, and reception/asylum shelters on health outcomes. This is especially important given that the length of stay in these settings can reach up to 15 years (36). In addition, a higher proportion of male individuals diagnosed with hypertension were observed in Denmark compared to Lebanon, which may reflect stress related to being unable to work, provide for their families, and meet gendered cultural expectations. Cultural and language barriers in Denmark are more pronounced than in Lebanon, and stress from navigating new systems and family separation may also contribute to the epidemiological profiles observed in this study.

Refugee health policies in both settings must prioritize lifestyle interventions to prevent cardiovascular disease morbidity and mortality. However, since Lebanon is an LMIC that is already struggling economically, policy changes to increase investments in NCD prevention and health promotion among refugees may be more feasible in Denmark. Denmark’s asylum seeker health policies need to be improved, especially in light of a growing body of evidence highlighting cardiovascular health disparities among resettled asylum seekers. Resettlement policies in Denmark have also resulted in a concentration of Syrian asylum seekers in low-income neighborhoods, which is associated with poorer cardiovascular and other health outcomes years after resettlement (37, 38). Adopting a human rights approach to the health of refugees and asylum seekers is critical for reducing disease burden and health disparities. In this context, this can be achieved by extending universal health coverage beyond acute care to include screening for hypertension, improving access to medications, facilitating psychosocial support, and ensuring equitable employment opportunities.

Our data also highlight the presence of barriers to care engagement in both Lebanon and Denmark, including difficulties accessing routine services such as hypertension management and medications. These barriers negatively impact the population’s cardiovascular health and contribute to preventable high medical expenditures. Healthcare access for migrants in general, and refugees and asylum seekers more specifically, varies from region to region. For example, 2016 data from the European Union (EU) Agency for Fundamental Rights indicate that among 28 EU countries, 14 provide suboptimal healthcare access, with free emergency care, but inaccessible pReemary and secondary care (39). A recent study conducted on 10 EU countries found suboptimal healthcare access for refugees, especially for chronic conditions, and called for renewed prioritization of refugee health access in Europe (40). In our study, none of the asylum seekers with hypertension in Denmark and only 1.6% in Lebanon were currently receiving antihypertensive medications. However, in Jordan, a country proximal to Lebanon that also hosts a lot of Syrian refugees, refugees are usually eligible for healthcare services through the Ministry of Health and other international organizations. Access to antihypertensive medications for refugees in Jordan is typically available, but refugees might need to go through a screening process to qualify for free or subsidized medicine. If they are not eligible, they may have to buy medication from pharmacies, which can be expensive for those without sufficient income. In Denmark, only acute care is offered to asylum seekers living in shelters as their applications are processed, which can often last several years. One study in Germany noted that despite high rates of mental illness among asylum seekers in reception centers and routine referrals to psychotherapy, none had received psychotherapeutic treatment months later (34). Such policies are common across Europe, resulting in delayed and inconsistent healthcare access for asylum seekers. Similarly, in Turkey, which has made significant efforts to integrate refugees, especially Syrian refugees, into its healthcare system, refugees without legal status or those not under the Temporary Protection program may struggle with access, especially to specialized care or medication care. Barriers to qualifying for access to pReemary care and antihypertensive medications remain in all of these refugee-hosting countries, contributing to disparities in hypertension prevalence and potentially increasing morbidity and mortality. Our findings highlight the need to expand universal health coverage for asylum seekers in Denmark and Lebanon and to strengthen the role of NGO involvement in hypertension screening, especially in Lebanon.

Our study has certain limitations. Data collection was hindered by the COVID-19 pandemic, changing border policies in Denmark, and political unrest in Lebanon. As a result, the study was forced to conclude before reaching the target sample sizes of 1,100 participants in Lebanon and 220 in Denmark. More than two-thirds of the respondents in Lebanon were women, compared to only half in Denmark, which might have slightly underestimated the overall prevalence of hypertension in Lebanon, as hypertension tends to be less prevalent in women (41). Another potential confounder is age, as the participants in Denmark were generally younger. Both age and sex differences are reflected in the higher prevalence of hypertension observed in Denmark, after adjusting for these and other potential confounders. Recall and respondent bias in surveys are also common; however, the survey’s structure—which asked the participants about diagnoses, symptoms, and medications—likely helped to prompt their memory of medical history. It is also possible that some respondents did not disclose health conditions out of fear that it might impact their asylum applications, although great care was taken during the consent process to assure the participants that study participation would not affect their immigration process. Only a few individuals refused to participate when invited—5% in Lebanon and 7% in Denmark. It was not possible to obtain detailed information on these individuals; however, the data collectors estimated that most of the “non-responders” were predominantly male individuals between 20 and 40 years of age, with no significant differences between Lebanon and Denmark. Finally, a one-time BP measurement cannot be used to confirm hypertension diagnosis; the prevalence rates based on the measured BP (rather than reported history or medication use) reported in this study were lower than those reported in previous studies, indicating that the true prevalence could be higher. A research design with follow-up would be able to investigate trends, estimate hypertension incidence, and potentially suggest causal relations. Although this was outside the scope of the present study, we are optimistic about such future projects.

## Conclusion

This study is among the first to document and compare the impact of recent displacement on hypertension among Syrian refugees along a migratory route in both LMICs and HICs. We highlight opportunities for intervention and policy development in both settings. The disparities in hypertension awareness and care were comparable in Lebanon and Denmark, despite Denmark having more resources to invest in refugee health. There is a need for longitudinal research tracking hypertension progression among refugees globally. Qualitative research is also needed to explore barriers to healthcare access and inform the design of behavioral and clinical interventions. More importantly, ensuring equitable access to antihypertensive medications and psychosocial support for refugees is essential to reducing preventable CVD morbidity and mortality.

## Data Availability

The original contributions presented in the study are included in the article/Supplementary material, further inquiries can be directed to the corresponding author.
