# Supplementary material for: Hypertension in Syrian refugees: prevalence, awareness, and access to care in Denmark and Lebanon
Source: Front Public Health. 2025 Jun 19;13:1486806. doi: 10.3389/fpubh.2025.1486806 (PMC12222061; doi:10.3389/fpubh.2025.1486806)
Supplement: Supplementary file 1 [file Data_Sheet_1.docx]

The analysis was stratified by host country and estimates of the prevalence and adjusted prevalence difference of hypertension was reported.


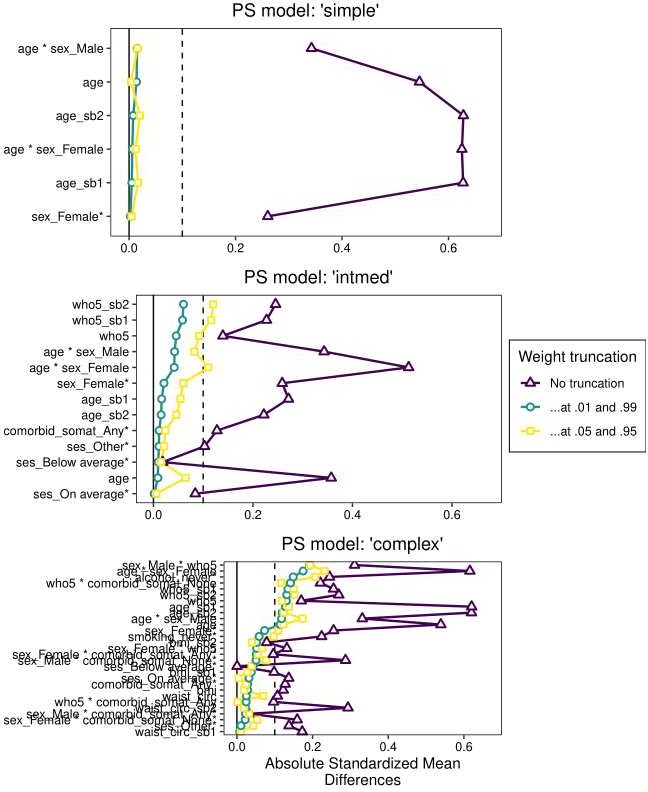


Abbreviations: Interact., interaction term; WHO-5, the WHO-5 quality of life scale; SES, socio- economic status; BMI, body mass index.

|  | **Enter** **as** | **Model** **1:** **“simple”** | **Model** **2:** **“intermedate”** | **Model** **3:** **“complex”** |
| --- | --- | --- | --- | --- |
| Age | 4-knots restricted cubic spline | x | x | x |
| Sex | Dichotomous | x | x | x |
| WHO-5 | 4-knots restricted cubic spline |  | x | x |
| Somatic co- morbidity | Dichotomous |  | x | x |
| SES | 4-level ordinal |  | x | x |
| Medication | 3-level ordinal |  |  | x |
| Alcohol use, actively | Dichotomous |  |  | x |
| Smoking, ac- tively | Dichotomous |  |  | x |
| BMI | 4-knots restricted cubic spline |  |  | x |
| Waist circum- ference | 4-knots restricted cubic spline |  |  | x |
| Sex & age | Interaction, no transformation | x | x | x |
| Sex & WHO-5 | Interaction, no transformation |  |  | x |
| Sex & somatic comorbidity | Interaction, no transformation |  |  | x |
| WHO5 & so- matic comor- bidity | Interaction, no transformation |  |  | x |

Covariates in each of the three propensity score models and how they enter. “Long-distance migration” is the response in all models. Abbreviations: Interact., interaction term; WHO-5, the WHO-5 quality of life scale; SES, socio-economic status; BMI, body mass index.

| Propensity score-weighted model | Prevalence difference  (95%CI) |
| --- | --- |
| Simple model*, multiple imputed | 5.0 (-4.7; 16.1) |
| Intermediate model*, multiple imputed | 4.2(-4.4; 13.5) |
| Intermediate model* with BP threshold 150/100, multiple imputed | 4.0(-4.7; 13.8) |
| Complete case, no adjustment | -6.8 (-16.2; 2.9) |
| Complete case, adjusted as the simple model* | -1.8(-11.2; 8.3) |
| Complete case, adjusted as the intermediate model* | 1.0(-9.0; 11.8) |

*Adjusted as indicated in Supplemental table 1. In all calculations Denmark is the reference: an adjusted prevalence difference of 5 indicates a 5 percentage point higher prevalence of hypertension in Denmark compared with Lebanon after confounding adjustment.

|  | Observed | | Multiple imputed | |
| --- | --- | --- | --- | --- |
|  | Lebanon | Denmark | Lebanon | Denmark |
| Age | 35 years old (IQR  = 20) | 30 years old (IQR  = 12.5) | 35 years old (IQR  = 20) | 30 years old (IQR  = 13) |
| Missing | n=58 (9.68%) | n=10 (8.8%) | NA | NA |
| Sex | Female: 72.73% | Female: 46.9% | Female: 72.37% | Female: 46.9% |
| Missing | n=38 (6.34%) | n=0 (0%) | NA | NA |
| WHO-5-score | 24 (IQR = 32) | 24 (IQR = 48) | 24 (IQR = 32) | 24 (IQR = 48) |
| Missing | n=47 (7.85%) | n=4 (3.54%) | NA | NA |
| Socioeconomic statusa | On average: 63.38% | On average: 50.68% | On average: 63.27% | On average: 52.04% |
| Missing | n=61 (10.18%) | n=≤40 (≤35%) | NA | NA |
| Alcohol use | Yes: 0.54% | Yes: 23.58% | Yes: 1.02% | Yes: 23.1% |
| Missing | n=46 (7.68%) | n=7 (6.19%) | NA | NA |
| Tobacco use | Yes: 27.39% | Yes: 50.46% | Yes: 27.65% | Yes: 50.09% |
| Missing | n=33 (5.51%) | n=4 (3.54%) | NA | NA |
| Waist circumference | 92cm (IQR = 23) | 96cm (IQR =  13.75) | 93cm (IQR = 22) | 95cm (IQR =  14.42) |
| Missing | n=58 (9.68%) | n=11 (9.73%) |  |  |
| BMI | 26.17kg/mZ (IQR  = 7.7) | 25.08kg/mZ (IQR  = 6.27) | 26.45kg/mZ (IQR  = 8.71) | 25.24kg/mZ (IQR  = 7.24) |
| Missing | n=64 (10.68%) | n=8 (7.08%) | NA | NA |
| Somatic comorbidity | None: 65.11% | None: 77.88% | None: 65.11% | None: 77.88% |
| Missing | 0 | 0 | NA | NA |
| Hypertension | Yes: 33.21% | Yes: 26.36% | Yes: 31.05% | Yes: 32.7% |
| Missing | n=51 (8.51%) | n=3 (2.65%) | NA | NA |
| Blood pressure, systolic | 120mmHg (IQR =  20) | 120mmHg (IQR =  24) | 120mmHg (IQR =  20) | 120mmHg (IQR =  24) |
| Missing | n=52 (8.68%) | n=3 (2.65%) | NA | NA |
| Blood pressure, diastolic | 78mmHg (IQR =  12) | 76mmHg (IQR =  16.75) | 78mmHg (IQR =  12) | 76mmHg (IQR =  17) |
| Missing | n=56 (9.35%) | n=3 (2.65%) | NA | NA |
